# New Approaches to Immunotherapy for HPV Associated Cancers

**DOI:** 10.3390/cancers3033461

**Published:** 2011-09-02

**Authors:** Anne-Sophie Bergot, Andrew Kassianos, Ian H Frazer, Deepak Mittal

**Affiliations:** The University of Queensland Diamantina Institute, Princess Alexandra Hospital, Brisbane, Qld 4102, Australia; E-Mails: andrew.kassianos@qimr.edu.au (A.K.); i.frazer@uq.edu.au (I.H.F.)

**Keywords:** HPV, vaccine, virus-like particles, immunotherapy

## Abstract

Cervical cancer is the second most common cancer of women worldwide and is the first cancer shown to be entirely induced by a virus, the human papillomavirus (HPV, major oncogenic genotypes HPV-16 and -18). Two recently developed prophylactic cervical cancer vaccines, using virus-like particles (VLP) technology, have the potential to prevent a large proportion of cervical cancer associated with HPV infection and to ensure long-term protection. However, prophylactic HPV vaccines do not have therapeutic effects against pre-existing HPV infections and do not prevent their progression to HPV-associated malignancy. In animal models, therapeutic vaccines for persisting HPV infection can eliminate transplantable tumors expressing HPV antigens, but are of limited efficacy in inducing rejection of skin grafts expressing the same antigens. In humans, clinical trials have reported successful immunotherapy of HPV lesions, providing hope and further interest. This review discusses possible new approaches to immunotherapy for HPV associated cancer, based on recent advances in our knowledge of the immunobiology of HPV infection, of epithelial immunology and of immunoregulation, with a brief overview on previous and current HPV vaccine clinical trials.

## 1. Introduction

Cervical cancer is the first cancer recognized by the World Health Organization (WHO) to be 100% attributable to an infection. Other cancers that are attributable to HPV infection include cancers of the vulva, vagina, anus and penis. The HPV family also includes the causative agents of genital and skin warts.

### Immunobiology of HPV & Epidemiology

Papillomaviruses are non-enveloped, double-stranded DNA viruses that infect the epithelium. Their genome encodes six non-structural proteins [Early 1 (E1), E2, E4–E7] and two structural proteins [Late 1 (L1) and L2]. Early proteins control the DNA-replication machinery of the infected cells by altering cell cycling and promoting viral genome replication, while late proteins, which form the capsid, are involved in the packaging of the virion [1].

The epithelium is a multilayered stratified tissue with a vertical organization correlated with keratinocyte differentiation and maturation: at the basement are dividing stem cells in contact with the dermis, followed by a layer of non-dividing keratinocytes, a squamous layer and finally, the mature squamous layer that is in contact with the external environment [2].

More than 100 HPV types have been identified, almost 40 of which infect the anogenital region. Genital HPV are divided into low- (*i.e.*, types -6 and -11, associated with anogenital warts and mild dysplasia), intermediate- and high-risk categories (*i.e.*, types -16, -18, -31, and -45, associated with high-grade dysplasia and anogenital cancers, such as cervical and anal carcinoma), depending on their association with cancer. The two most studied HPV are HPV-16 and -18, which contribute to almost 70% of all cervical cancers. Three novel papillomaviruses, HPV-109 from an SCC, HPV-112 from a condyloma and HPV-114 from a cervical lesion, have been identified recently, with more HPV types still to be characterized in HPV-associated lesions [3].

Genital papillomaviruses infiltrate epithelial lesions and infect cells of mucosal or squamous epithelia. As long as the infected epithelial cells are dividing, the virus will remain in its free (non-integrated) episomal form and replicate using the cellular DNA-replication machinery. At an early stage, the proteins E1 and E2 delay cell differentiation and promote cell replication and thus viral genome amplification. When epithelial cells mature and stop dividing, virion particles are formed and mature virions can be released from the epithelial cells. HPV-induced genital lesions often regress spontaneously within a year, but may persist in about 2% of cases for five years or more, with the histological hallmarks of CIN1 (cervical intraepithelial neoplasia). As the infection persists, these lesions can progress to CIN2 and CIN3. In one-third of cases, CIN3 lesions can develop into cancer. In cervical cancer lesions, the HPV DNA is mostly found integrated in the cellular genome [1].

## 2. Immune System and HPV

### 2.1. Role of the Immune System: Lessons from Immunocompromised Patients

The incidence of some cancers is higher in immunocompromised patients. Immune suppression after organ transplantation is connected with increased risk of virus-associated cancers [4] including HPV-associated cancers. For example, individuals with acquired immunodeficiency syndrome (AIDS) are at higher risk of HPV-associated cancers [5,6], consistent with the higher prevalence and more frequent persistence of HPV infections [7,8]. HPV-induced anal cancer in an HIV-positive population is most commonly associated with HPV-16 infection (75%) followed by HPV-18 (less than 10%). The prevalence of HPV in anal cancer is higher in women (90%) than in men (75%) [8].

Immunosuppression is often reflected in low CD4 T-cell numbers in individuals with HIV. In a recent study, data from individuals diagnosed with AIDS over twenty five years were collected and analysed for HPV–associated cancers. A low CD4 T-cell count measured at AIDS onset was associated with significantly increased risks of HPV-associated invasive cancers, including anal cancers among men and vaginal or vulvar cancers among women. In the same study, in situ cervical cancer risk was lower and invasive cervical cancer risk was higher in patients with AIDS, although differences were not statistically significant due to the lack of sufficient data for cervical cancer patients. In contrast to the high risk of anal and other HPV-associated cancers, the risk of invasive oropharyngeal cancer was only moderately elevated among persons with HIV when compared with the general population. This modest increase in risk may reflect the fact that only a few oropharyngeal cancers (about 30% of tonsillar epithelial cancers) are caused by HPV [8] or the longer time for progression of HPV infection in the mouth to cancer. Although providing a link between immune suppression (low CD4 T-cell count measured at AIDS onset) and increased incidence of HPV-associated cancers, the above-mentioned study did not consider HIV burden at the time of AIDS onset, which is another marker of the degree of immunosuppression and may be important in the natural history of HPV-associated cancers [8]. The evidence discussed above thus suggests a direct role for the immune system in the control of infection by HPV.

### 2.2. Role of the Immune System: Spontaneous Immune Responses to HPV-Associated Cancer

Most HPV infections are transient, becoming undetectable within the first two years in immunocompetent individuals. While the spontaneous regression of latent HPV infection is difficult to assess due to the sensitivity of assays and challenge in discriminating between positive and negative samples (discussed in 3.2), lesions have been shown to often regress spontaneously in young women [9,10]. It has also been reported that a few percentage of CIN2-3 lesions can regress spontaneously to CIN1 or less [11]. However, different spontaneous regression rates exist depending upon the type of HPV, with high-risk HPV-16 and phylogenetically linked HPV being less spontaneously cleared than low-risk HPV [12,13]. The spontaneous regression of HPV lesions is associated with the local presence of a range of adaptive immune cells (B cells and CD8+, CD4+ and CD25+ T cells) [11,14]. Data from immuno-deficient or -competent individuals suggest that the immune system is highly involved in the success or failure of spontaneous HPV clearance. This also implies that boosting such immune responses by therapeutic vaccine could enhance clearance rates. In the next paragraphs, we will discuss the role of immune cells during HPV infection.

### 2.3. Role of Immune Responses and Cell Types with Regards to HPV

#### 2.3.1. Humoral Immunity

Adaptive immune responses to HPV infection include humoral (B cell, antibody) responses specific for viral structural (L protein) or non-structural (E protein) antigens. The L1 major structural protein of HPV assembles into particles or capsids indistinguishable from native virions. Although papillomavirus capsids have been shown to be quite immunogenic when injected into animals [15,16], the natural antibody response to L1 is weak, probably because the L1 protein is expressed in the squamous epithelium, a site that may not allow efficient immune activation. Nevertheless, those capsids are a major tool for the study of serological responses to HPV. Most studies have examined the prevalence of antibodies to HPV capsids by ELISA [17] and compared their findings with the presence of HPV DNA using PCR-based testing and neoplasia or genital warts by Papanicolaou (Pap) screening.

There is considerable variability in the timing of the HPV-specific antibody response in relation to HPV infection. Nevertheless, serum IgG antibodies, which have a neutralizing potential, develop in response to infection in more than 50% of women. The kinetics of antibody development are slow (6 to 12 months), and the peak titres are low, but antibodies can persist for decades unless HPV-associated lesions resolve. Secretory IgA antibodies can also be detected in the cervical secretions of HPV-positive women with a similar time course of appearance to IgG antibodies, but with a shorter persistence time [18]. However, studies have also suggested that antibodies to HPV may persist for years after the disappearance of measurable disease, making it difficult to discriminate whether antibodies are due to past or persistent infection [19,20]. Antibodies to non-structural proteins after natural infection are rare or nonexistent. However, relative to the presence of HPV DNA at the initial stages of infection, antibodies against HPV-16 and -18 non-structural E2, E6 and E7 proteins were detected in only half of patients tested [21]. A stronger correlation has been demonstrated between E7 antibodies and advanced cervical cancer [22-24].

**Figure 1.**
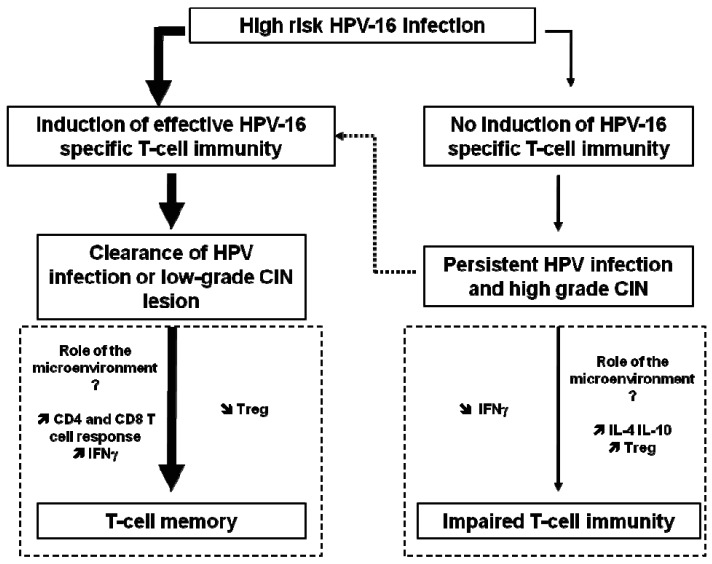
Proposed model for the association between HPV-16-specific T-cell immunity and the development of disease (adapted from [26]). *Thick arrows* represent the fate of the majority of HPV-16-infected individuals, in contrast to *thin arrows* that represent the fate of the minority of HPV-16-infected individuals. *Dotted arrow* indicates that some cases of spontaneous regression can occur, probably by the induction of T-cells and/or cells from the microenvironment, which leads to the destruction of infected tissues or tumor mass. *Dashed box* represents the immunological mechanism that is likely involved.

HPV antibody assays are problematic because the levels of antibody are low, the threshold for positivity is difficult to define, and correct antigens (with the exception of L1 VLP) are not well characterized. Spontaneous clearance of HPV infection occurs in the majority of patients, and has been largely attributed to the cellular immune response rather than the humoral immune response, as further described below and in Figure 1.

#### 2.3.2. Cellular Responses

##### CD4+ and CD8+ T-cells

High-risk HPV infections can progress to high-grade CIN due to impaired T-cell immunity (Fig 1). HPV early proteins are required early in viral infection and therefore may serve as a useful vaccine target in HPV-infected individuals, aimed at prevention or therapy of premalignant lesions. Since the E6 and E7 oncoproteins of HPV viruses are expressed in precancerous lesions, such proteins might be potential tumor-specific targets for immunotherapy. The E5 protein of HPV-16 is known to downregulate MHC class I expression on infected cells and could be targeted to allow re-expression of MHC and recognition of infected cells by CD8+ T cells [25].

There are several studies providing information about CD4+ and CD8+ cytotoxic T-cell responses to HPV-16 early proteins [27]. Using fusion proteins, panels of overlapping peptides, virus-like particles or tetramer technology, CD4+ T cell responses to HPV-16 E2 [28], E4 [29,30], E5 [31], E6 [29,32] and E7 [33] have been demonstrated in both patient and healthy control populations without clear consequences for resolution of HPV infection. HPV-16 E2-, E6- and E7-specific CTLs can also be detected in patients with previous or ongoing HPV infections [34,35]. Moreover, T-cells can also be found that recognize late proteins, such as HPV-16 L1 [36], although responses to L1 are not thought to be important in viral clearance once HPV infections are established. Overall, HPV specific T-cells detected in patients with HPV infection are not anergic but functionally active, as has been demonstrated using a variety of restimulation protocols such as gamma-interferon (IFN-γ) release by ELISPOT and chromium 51 release by cytotoxic assay [34,35]. To highlight this, a recent study described the first clinical success for therapeutic vaccination of vulvar intraepithelial neoplasia (VIN)3 patients with a vaccine which comprised HPV-16 E6 and E7 synthetic long overlapping peptides (HPV16 SLP). This vaccine induced a CD4+ and CD8+ T cell response with IFN-γ production and led to a durable and complete regression of HPV-induced lesions in half of the patients [37].

##### NKT

Natural killer T (NKT) cells are a heterogeneous group of T cells that recognize self or microbial lipid antigens presented by CD1d molecules. CD1d is a major histocompatibility complex (MHC) I-like glycoprotein present on the surface of not only antigen presenting cells but also intestinal epithelial cells, keratinocytes and reproductive tract epithelial cells [38].

The most widely studied ligand of NKT cells is α-galactosylceramide (α-GalCer) [38]. A recent study has used α-GalCer as an adjuvant with DNA vaccination against HPV-16 oncoprotein E7 to generate high numbers of E7-specific CD8+ T-cells protective against the transplantable TC-1 (HPV-16+) tumour model in mice [39]. Another NKT ligand, β-galactosylceramide, has also been shown to be protective against the same tumour [40]. These data thus highlight the potential of NKT ligands as therapeutic agents for HPV-associated cancers.

MHC cell surface downregulation is one of the mechanisms employed by a variety of tumours to evade immune detection. Cell surface CD1d, a MHC-Ib molecule, is down-regulated in HPV-related lesions and in HPV-negative cervical cancer cell lines stably transfected with HPV-6 E5 and HPV-16 E5, thus linking decreased CD1d expression in the presence of HPV infection with evasion of NKT cells [41]. On the other hand, studies in a mouse model expressing HPV-16 E7 oncoprotein in keratinocytes have shown that IFN-γ produced by invariant NKT cells in the skin provides protection against E7 transgenic skin graft rejection [42]. A small study on a North Indian population has also shown an association of the *IFN-γ* +874 polymorphism with an increased risk of cervical cancer in patients at stages III + IV. However, this study did not include all clustered polymorphism sites of *IFN-γ* and was not free from selection bias [43].

Based on the literature, NKT cells may play two different roles, depending on the type of HPV lesions. In established HPV-associated cancerous lesions, activation of NKT cells may provide therapeutic value, while inhibition of NKT cell activity or recruitment in precancerous lesions may allow a protective immune response.

##### Treg

Regulatory T cells (Treg) are CD25hi Foxp3+ CD4+ T cells that have been involved in the failure of the immune system to control the development of numerous cancers both in humans and in mice [44,45]. Therefore, a high frequency of Treg in human HPV-associated cancer could counteract the host immune response and thus influence therapeutic strategies.

Indeed, human, high-grade CIN lesions (CIN3) and cervical carcinomas have been shown to contain higher numbers of infiltrating lymphocytes and FoxP3+ Treg compared to colon carcinomas, skin melanomas, and bronchial carcinomas [46-48]. Moreover, mucosal enrichment of Treg cells was associated with a diminished cellular immunity in the cervical mucosa, and both seemed to contribute to the development of high-grade CIN 2 and 3 lesions [49].

Notably, Treg may also be induced by therapeutic vaccines. In a study comparing patient groups with small or large HPV-16-positive vulvar lesions, larger lesions were found to contain higher frequencies of vaccine-induced HPV-16-specific Foxp3+ Treg cells, correlating with early treatment failure [50]. A similar result has also been observed in a mouse model [51]. Moreover, it has been shown that the process of repeat vaccinations can increase the presence of regulatory T cells [52]. This suggests that the combination of therapeutic vaccination with the depletion of Treg or at least the abrogation of their suppression should improve cancer therapy.

The *in vivo* depletion of Treg has already proved to be efficient in allowing the rejection of established tumours in mice and humans [53-58]. In an animal model of HPV-associated cancer, the therapeutic depletion or inactivation of Treg has been shown to induce a strong intratumoral invasion of CD8+ T cells and complete eradication of HPV-16 E6/E7-expressing tumor cells in 70% of treated animals [48]. Moreover, combining T-cell therapeutic vaccination and Treg depletion can lead to the complete eradication of HPV-expressing tumors in mice [59]. In patients with condylomata acuminata, Treg depletion using cyclophosphamide ameliorates the immune milieu of the lesion site, leading to the elimination of remnant viruses and helps prevent recurrence after laser therapy [60]. As a consequence, the role of Treg should be scrutinized within HPV vaccination strategies. The temporary removal of Treg from patients should be considered as a means to improve the benefits of therapeutic vaccination targeting HPV-associated lesions.

#### 2.3.3. Myeloid Cells

##### Macrophages

Macrophages are one of the three types of phagocytes in the immune system and are distributed widely in the body tissues, where they play a critical part in innate immunity. Macrophages are found in many areas of lymph nodes, particularly in the marginal sinus and in the medullary cords. Here they can actively phagocytose pathogens and antigens, and so prevent them from entering the blood. Activated macrophages undergo changes that greatly increase their antigen-presenting function and anti-pathogen effectiveness, and amplify the immune response.

Regarding their role in HPV infection, studies in women infected with HPV have shown a positive correlation between lesion grade, the number of infiltrating macrophages and IL-10 expression by these cells [61-63]. However, macrophages have also been reported to react against E6 and E7. Macrophages can kill HPV-16 E6 but not E7-expressing tumor cells through tumor necrosis factor (TNF)-α and nitric oxide (NO)-dependent mechanisms [64,65]. It has also been shown that HPV-16 E7, but not E6, NIH-3T3 human cells were susceptible to activated macrophages, and that the ability of E7 to cause transformation was required to induce susceptibility of infected cells to activated macrophages [66]. Thus, the role of macrophages in HPV infection is still unclear.

The tumor microenvironment has been shown to influence tumor immune privilege, and this has become a new field of research in tumor immunology. Macrophages are enriched in several types of human cancer, including breast, ovarian, non-small cell lung cancer, and Hodgkin's lymphoma, and their presence correlates with a poor clinical outcome. Tumor-associated macrophages (TAM) have been therefore identified as regulators of tumor development. As an example, TAM have been shown to play a major role in colorectal cancer by recruiting Treg to the tumor mass, thus favouring an immunosuppressive microenvironment that leads to tumor growth [67]. TAM can also act by suppressing tumor-infiltrating T-cells through several mechanisms, as seen for example under hypoxic conditions [68]. TAM have been shown to be recruited *in situ* through the macrophage colony-stimulating factor (CSF-1), as its over-expression correlates with poor prognosis in breast cancer [69]. Therefore, macrophages can have opposing roles within the immune response against tumorigenesis and infection, and their role in high risk HPV infection is yet to be investigated.

##### Myeloid derived suppressor cells

Myeloid derived suppressor cells (MDSC) are a heterogeneous population of cells that have been associated with cancer, inflammation and infection, and been shown to suppress T-cell responses. A regulatory role for MDSC, as well as regulatory T cells, has been highlighted in animal models using transplantable tumour cells expressing the E7 protein, TC-1. One recent study found reduced MDSC and Treg numbers in both the spleen and transplantable tumour itself through the use of a tri-therapy combination HPV vaccine, leading to the restoration of a potent E7-specific CD8 T-cell response and the control of tumour growth [70]. Another study using the same tumour line, but E7 DNA vaccine instead, showed a reduction in MDSC numbers but not Treg cells in the tumour microenvironment, which was sufficient to control tumour growth. Adding Imiquimod to the E7 DNA vaccine further reduced MSDC numbers and activated of a CD8 response, improving the anti-tumour response mediated by E7-specific CD8 T cells, macrophages and NK1.1+ cells [71].

Despite no demonstrated association with HPV-associated cancers in humans, MDSC have been observed with increased prevalence in the peripheral blood and tumor microenvironment of patients with head and neck squamous cell carcinomas [72] and pancreatic cancer [73]. By further elucidating the mechanisms of MDSC recruitment and maintenance in the tumor environment in mice and humans, new vaccine strategies may be developed to reverse the suppression of anti-tumour immunity.

##### Mast cells

Mast cells (MC) are localized at body sites that interface with the environment, such as the skin and mucous membranes, which are also the site of HPV infection. Mast cells are highly specialized innate immune effector cells that contain secretory granules in which large amounts of proteases are stored in complexes with serglycin proteoglycans [74]. The presence of mast cells along with other immune cells has been shown in CIN2/3 lesions [75], though their main role in HPV pathogenesis is difficult to determine due to their “tunable” function. They can indeed act as pro-inflammatory cells through the recruitment of innate and adaptive immune cells, or as immuno-suppressive cells through the production of the immunosuppressive cytokine IL-10 [76]. Burns [77], tape stripping, tumors [78]*,* allergy, parasitic and virus (HIV) infections have each been shown to recruit mast cells into the skin.

A major point linking mast cells to cancer is that mast cells accumulate in the stroma surrounding certain tumors, especially mammary adenocarcinoma, where they have been shown to synthesize and secrete potent angiogenic cytokines, such as vascular endothelial growth factor (VEGF). These molecules facilitate tumor vascularisation by a direct angiogenic effect and by stimulating the stroma and inflammatory cells of the tumor microenvironment [79,80]. In relation to their ‘tunable’ function, mast cells in the local environment can also be detrimental for the tumours themselves, secreting immune mediators such as IL-1, IL-4, IL-5, IL-6 and TNF-α that can induce apoptosis of tumor cells and recruit inflammatory cells [80]. As is the case for macrophages, the role of mast cells in the immune response to HPV infection is still largely unknown.

##### Dendritic cells

Dendritic cells (DC) are potent antigen-presenting cells (APC) that play a fundamental role in the induction and regulation of innate and adaptive immune responses against microbial pathogens. DC in human and mice can be broadly categorised into two major populations: plasmacytoid DC (pDC) and conventional DC (cDC), which can be further divided into migratory DC (Langerhans cells (LC) and interstitial DC) residing in peripheral tissues and lymphoid tissue-resident DC.

In humans, pDC are present in cervical cancer lesions, primarily in the stroma underlying the tumor rather than the dysplastic epithelium, and produce the anti-viral cytokine, IFN-α, in response to HPV VLP [81,82]. These studies suggest pDC play an important function in the natural immune response against HPV, although their role in cervical cancer development remains unclear.

Epithelial LC are the model migratory DC, initiating immune responses to infecting pathogens by capturing Ag and delivering it to the T cell areas of the draining lymph nodes. Several studies have documented reduced numbers of LC in human HPV-associated cervical lesions [83-86], suggesting the depletion of epithelial LC during HPV infection may lead to prolonged infection and possibly oncogenesis. Furthermore, LC incubated with HPV VLP fail to up-regulate surface activation markers or initiate a HPV-specific immune response, suggesting that HPV avoids immune recognition through poor stimulation of LC [87, 88].

A unique population of interstitial DC has been recently defined in the cervical stroma, with elevated numbers of these cells in human cervical cancer relative to normal cervix. This stromal DC subset is distinct from LC and expresses immuno-suppressive factors IL-10, TGF-β and indoleamine 2,3-dioxygenase (IDO) [63,75,89]. However, there are several additional mechanisms by which stromal inflammatory cells could contribute to tumor escape. For example, PD-L1 (B7-H1), expressed on multiple cell types including APC, and PD-L2 (B7-DC), selectively expressed by DC and macrophages, are both expressed in human cervical cancers [90].

DC are regarded as the master regulators (chef d'orchestre) of the immune response. They have been utilized in numerous vaccination protocols to boost antigen presentation and T-cell costimulation and thus, augment antigen-specific T-cell responses. However, their multiplicity of phenotypes and functions make it difficult to know which population(s) of APC should be targeted.

## 3. Prophylactic HPV Vaccines

### 3.1. VLP

Virus-like particles (VLP) are a useful tool for development of vaccines and are often used in studies to identify viral assembly proteins. They resemble the native virus immunologically but are non infectious as they don't contain viral genetic material. Two prophylactic HPV vaccines (Cervarix and Gardasil) that are currently in use are based on the L1 major capsid protein. The HPV L1 open reading frame translates a 55-kD protein that efficiently self assembles into viral capsomeres and empty capsids, when expressed in eukaryotic cells [91]. In papillomavirus VLP vaccine production, the L1 gene is cloned, and amplified using primers specific for the L1 gene. The amplified L1 segment is then inserted into an appropriate intermediate expression vector and used to produce recombinant L1 yeast [92], vaccinia [91] or baculovirus [93]. These are then purified and used to express L1 in eukaryotic cells [91]. The L1 self assembles to form VLP and does not require L2 or other non structural proteins. The resulting VLPs are immunologically similar to the native viron. These are then finally combined with alum-based adjuvants to make the basis for prophylactic vaccines.

#### Cervarix & Gardasil: 5-years overview

Cervarix™ is a bivalent vaccine manufactured by Glaxo-SmithKline that incorporates two HPV types (HPV-16, HPV-18) responsible for 70% of cervical cancers. Gardasil™, manufactured by Merck, is a quadrivalent vaccine that additionally incorporates HPV-6 and HPV-11. HPV-6 and -11 cause an estimated 75-90% of genital warts cases.

#### Cervarix

HPV-16 and -18 VLP are produced in Trichoplusia ni Rix4446 cell substrate using a baculovirus expression vector system and formulated with the adjuvant system ASO4 that is comprised of 3-O-desacyl-4′ monophosphoryl lipid A (MPL) and aluminum hydroxide salt. The vaccine is administrated intramuscularly in three dose schedule - months 0, 1 and 6.

#### Gardasil

HPV -6, -11, -16 and -18 VLP are produced in yeast *Saccharomyces cerevisiae* and formulated with adjuvant aluminum hydroxyphospate sulfate. The vaccine is administrated intramuscularly in three dose schedule - months 0, 2 and 6 [79].

These prophylactic vaccines are licensed for use to prevent HPV infection and anogenital cancers in females and males aged 9–45 in many countries, and are administrated to teenage girls as a part of the routine immunization schedule in some. In addition, the quadrivalent vaccine is licensed for use to prevent genital warts in some developed countries. The use of these vaccines in developing countries is limited by cost-related issues. Thus, provision of prophylactic HPV vaccines to a wider population through development of biosimilar vaccines is under consideration in developing countries.

As long as women are sexually active they remain at risk of cervical HPV infection. Therefore, it is essential that HPV vaccines provide long-lasting protection. Clinical efficacy has been observed up to 6.4 years for the bivalent vaccine [94] and up to 8.5 years for the quadrivalent vaccine [95,96]. Since most HPV infections are silent, it will be many years before we know for certain about the duration of protection and efficacy provided by HPV vaccines. A mathematical modeling study based on three different statistical models and follow-up data from more than 300 vaccinated women, has predicted that anti-HPV-16 and -18 antibody levels with the bivalent vaccine will persists for at least 20 years [94]. In addition to showing 100% efficacy in preventing pre-cancerous lesions, HPV vaccines also provide some cross-protection against other HPV subtypes (60% and 78% efficacy in preventing incident infections secondary to subtypes HPV-31 and -45 respectively) (reviewed in [97]).

Based on phase III clinical trial results [98], the Advisory Committee on Immunization Practices (ACIP) states that women should be advised that the vaccine will not have any therapeutic effect on existing infection or disease and they should continue to receive routine cervical cancer screening [99].

### 3.2. Who and When To Vaccinate

To allow meaningful approaches to vaccine development, both prophylactic and therapeutic, it is important to identify the age at which the population is infected, the extent of exposure to human papillomavirus within the general population, how long the infection persists and if the immune response to HPV is consistent throughout life. One of the key questions also remains to understand the first transmission of the virus and whether sexual transmission is the only route for high-risk HPV.

#### Perinatal transmission

The detection of human papillomaviruses including HPV-16 and -18 in neonates have been described in about 50% of cases where the mother is infected at the time of delivery. In two early studies, HPV-16 and -18 DNA were detected in buccal and genital swabs in more than 70% of infants at 24 hours post delivery and persisted in more than 80% of contaminated infants at 6 weeks through to 6 months of age [100,101]. Other studies also provide evidence that HPV-DNA could be found within the nasopharyngeal aspirate fluids and oral cavity of neonates [101-104].

The mode of delivery at birth, vaginal *versus* caesarean, may play a role, as a significantly higher rate of HPV-16/-18 infection was found at birth when infants were delivered vaginally [105]. Ten years later, two studies analysed the type-specific HPV concordance in infected mothers, the placenta and the newborn or the infected mother and the cord blood in about 60 to 70 cases, but they only found a small incidence of placental infection, and an even smaller incidence of transplacental transmission [106,107]. One of the largest studies of newborn HPV infections to date, and the first to use sequencing methods to evaluate if a vertical transmission of HPV from infected parents does occur, showed a lack of concordance for the HPV type between the mother and child or the father and child [108]. These data support the rarity of perinatal high-risk HPV transmission and suggest other potential sources of exposure or contamination. Altogether, there is no obvious evidence for the transmission of high-risk HPV-16 and -18 to the genital tract at birth. Although common to detect HPV-16/-18 DNA in the oral cavity of newborns, it does not persist nor replicate, and the only disease attributable to vertical transmission is for recurrent respiratory papillomatosis (RRP), which is caused by HPV types -6 and -11 with a 1:1000 live birth event [95].

#### Adolescence

If the only possible method of genital HPV infection is sexual transmission, non-infected individuals could be found within virgin girls and young women. On the other hand, if nonsexual transmission can occur, some virgins might have genital HPV-specific antibodies, and this would increase the cut-off and reduce test sensitivity if virgin females were used to provide a reference standard for antibody testing. Therefore, it would be informative to determine the antibody status in pre-pubertal children. Here, the results from different studies are controversial, as it is crucial to define the antibody detection methodology, and what would be a “positive” and a “negative” control for an assay.

One large study in Edinburgh examined more than 1,000 serum samples from a cohort of 11–13 year-old virgin school girls for the presence of antibodies to HPV-1, -2, and -16 VLP. The study reported that 7% of study subjects had antibodies to HPV-16 VLP, 52% to HPV-1 and 38% to HPV-2. In contrast, a Swedish study showed that virgin teenage girls were not seropositive in an HPV-16 VLP-based ELISA, while 14% of early sexually experienced girls were detected HPV seropositive and positive for HPV-16 DNA, leading to a conclusion that non-sexually transmitted infections are rare or nonexistent among adolescent girls [109]. Another study conducted in South Africa with children aged between 1 and 12 years found that 4.5% of sera tested were positive for antibodies to HPV-16, with a prevalence decreasing with age. This could indicate vertical transmission of HPV infection, but HPV DNA from children and parents were not tested to confirm or invalidate this conclusion [110]

Altogether these data show the difficulty in defining an HPV antibody positive from a negative serum. This makes interpretation of data on early HPV infection problematic. However, the balance of evidence suggests that there are only very few, if any, HPV infections caught before the onset of sexual activity.

#### Adult age

The prevalence of high risk HPV in the general population is well known. Women acquire HPV infection soon after onset of sexual activity [111] and more than 50% of adult women are HPV-16 seropositive, a percentage increasing with the number of partners and sexual behaviour. Moreover, studies on older women (>65 years old) indicate an impairment in host immunologic responses, with decreased lymphoproliferative responses associated with persistent HPV infection [112,113]. Taken together, the available data lead to the conclusion that the HPV prophylactic vaccines currently available should ideally be administered before the onset of sexual activity, prior to exposure to HPV infection.

#### Men

Cancers of the penis, anus, and oropharynx in males can be due to high risk HPV infection, although less than 25% of HPV-related cancers occur in males. However, HPV infections and related non-malignant diseases are common in males [114, 115]. The rate of genital HPV infection in males, and the probability that a sexually active male will acquire a new genital HPV infection, are equivalent to the rates in females. Increasing numbers of sexual partners and preference for male partners are each associated with an elevated risk of HPV acquisition [116]. However, there are differences between the sexes in the immune response to genital HPV infection: seroprevalence in men is lower than in women (7.9%, *vs.* 17.9%, respectively), with lower titres of antibodies [114,117].

A recent study enrolled more than 4,000 healthy boys and men between 16 to 26 years of age from 18 countries in a randomized, placebo-controlled, double-blind trial, and demonstrated that the quadrivalent HPV vaccine used as a prophylactic vaccination can reduce the incidence of some HPV-related infections [118]. There are two potential advantages to vaccinating males: the first is the direct benefit for those immunised and the second, indirect but not less important, is that this can improve protection of females, by reducing virus transmission.

Despite the success of preventive HPV vaccines, such vaccines are unlikely to reduce the global burden of HPV-associated cancers in the next few years due to their high cost and limited availability in developing countries, where there is a high incidence of cervical cancer. Moreover, existing preventive HPV vaccines do not generate therapeutic effects. Therefore, it is worthwhile to consider alternative strategies to treat HPV-associated premalignancy and malignancy.

### 3.3. When To Vaccinate with a Therapeutic Vaccine? The Sooner, the Better…

#### Thymic involution with age

With age, the immune system undergoes dramatic changes. From fetal to neonatal periods, naïve T-cells are extensively produced and migrate from the thymus to secondary lymphoid organs in the periphery, where they accumulate and display a broad and polyclonal repertoire. From youth to adulthood, thymic export is balanced by cell death at the periphery, leading to an equilibrium in the apparent quantity of cells. With age, the thymus involutes and numbers of newly generated naïve T-cells gradually fall. Together with this thymic involution, an immune decline, termed immunosenescence, progressively alters B- and T-cell functions. In counterpart, an accumulation of antigen-experienced peripheral T-cells is observed, both in the CD4+ and CD8+ compartments, likely due to an accumulation of memory responses following antigen activation and from homeostatic proliferation to maintain T-cell levels [119]. It is reported that the TCR repertoire of elderly persons (age > 75 years) is severely contracted. In fact, the TCR diversity of elderly persons in both the CD4+ and CD8+ compartments is at least 100-fold less diverse than the TCR diversity in younger individuals (age 20–35) [119,120]. As a consequence, it seems that a therapeutic vaccine should be administered early enough during adulthood to elicit the maximum efficiency.

#### Critical window of time

Another fact arguing in favour of an early therapeutic vaccination comes from the analysis of vaccination strategies against transplantable tumours in mice. Indeed, it has been shown in mice that a vaccine is more potent and will generate a strong anti-cancer effector response when it is administered within a certain time frame [121]. In humans, in HPV-16-positive vulvar lesions, it has been shown that patients with a smaller lesion and a shorter history of disease were significantly more responsive to therapeutic vaccines, compared to patients with larger lesions and longer history of disease [50]. This suggests that the earlier a therapeutic vaccine is administered after the appearance of cancer, the improved probability of a positive clinical response.

#### The side effects: Treg cells

We have previously discussed in Section 2 the deleterious role of Treg in cancer. Moreover, it has been described in mice that, at the very time of tumour emergence, self-specific Treg were activated early and briskly by self-antigens expressed by tumours, driving a secondary-type immune response in essence more rapid and efficient than the primary-type response of naive effector T-cells specific for tumour neoantigens [55]. This mechanism of ‘déjà vu’ explains an old paradigm of cancer immunology, that preventive immunization is more effective than therapeutic immunization.

Therefore, here again, it seems that early vaccination after the detection of cancer might be the preferred option to promote a maximum anti-tumour response. In that case, an improved effort in the screening of cervical cancers or skin cancers induced by HPV infection is also a key in the success of immunotherapy for HPV-associated cancers.

## 4. Therapeutic HPV Vaccines

Current treatments for HPV-associated lesions rely primarily on excision or ablation of the infected lesion. Ablative therapies are effective when the disease is localized as in the case of CIN but the possibility of recurrence remains, as treatment does not always eradicate the underlying HPV infection. A successful immunotherapy might therefore be a preferred mode of treatment because it can target all HPV-associated lesions irrespective of their location. Ideally, it would also induce long-lasting immunity, thus preventing recurrence.

Therapeutic cancer vaccines are anticipated to treat an existing cancer by enhancing the naturally occurring immune response to the cancer. Unlike prophylactic vaccines, which protect against HPV infection by generating neutralizing antibodies, therapeutic vaccines would likely require induction of antigen-specific T-cells for clearance of HPV-associated lesions (reviewed in [122]). Most of the therapeutic vaccines developed to date for HPV-associated disease have failed at the clinical stage, despite promising results in preclinical animal models. One possible reason for failure is that these vaccines have generally been tested in advanced stage cancer patients, where the chances of their success are reasonably poor. Nevertheless, we have to mention here some of the recent and successful therapies against HPV-associated cancers, in HPV-16 associated vulval intraepithelial neoplasia [37,123] and in HPV-16 associated cervical cancer [124]. They will be further discussed in each section of this chapter. The therapeutic vaccine should also be directed against the right target. The capsid proteins L1 and L2 are no longer expressed after the integration of HPV DNA into the DNA human host cells, rendering them inappropriate as therapeutic targets. On the contrary, the two oncoproteins E6 and E7 are expressed throughout the viral life cycle and are required for continued tumor growth. The E6 oncoprotein degrades tumor suppressor p53 via direct binding to the ubiquitin ligase E6AP and contributes to tumorigenesis [125,126]. The E7 oncoprotein binds and induces proteasomal mediated degradation of the retinoblastoma family of proteins (pRB, p107 and p130), which are required for processes such as cell cycle progression, DNA repair, apoptosis, senescence and differentiation [126, 127]. Hence, E6 and E7 oncoproteins appear to be good candidates for HPV vaccination strategies. However, the proteins E1 and E2 are expressed early in the progress of an HPV infection before the integration of the viral genome into the host DNA. E1 encodes for the helicase and E2 is a regulatory protein and they both have a role in viral replication. In line with our hypothesis that the vaccine should be delivered as soon as possible after infection by HPV (as discussed in Section 3.3), E1 and E2 may be the best targets for a therapeutic vaccine. Indeed, vaccination against E1 and E2 have already been shown to induce protection in dogs [128] and rabbits [129]. In humans, E1 and E2 have been reported to induce both natural and vaccine-induced T-cell responses in patients with persistent cervical neoplasia [130]. Together with a good target, HPV vaccines need to be delivered correctly. In the following sections, we will discuss various strategies as candidates for delivery of therapeutic HPV vaccines.

### 4.1. Live Vector-Based Vaccines

Live-vector based vaccines are highly efficient in delivering relevant antigens or DNA encoding antigens of interest. These vaccines have advantages including the possibility of choosing a desirable vector from a wide range of vectors to deliver antigens. Moreover, replication and spreading of live vectors in the host results in potent immune responses. However, using live vectors poses safety concerns in clinical applications. Additionally, neutralizing antibodies to the live vector, generated upon vaccination, may limit the efficiency of repeated immunizations with the same vector. The most common types of live vectors used for vaccinations are bacterial and viral vectors.

#### 4.1.1. Bacterial Vectors

The two most promising bacterial vectors for therapeutic HPV vaccines are *Listeria* and *Salmonella*. *Listeria* is a gram-positive intracellular bacterium that occasionally causes disease in humans. *Listeria monocytogenes* (LM) has the ability to replicate in the cytosol of APCs after escaping phagosomal lysis by secreting a factor called listeriolysin O (LLO). This unique feature allows peptide antigens derived from LM to be processed and presented via both MHC class I and class II pathways, resulting in potent CD4+ and CD8+ T-cell-mediated immune responses.

##### Preclinical models

*Listeria*-based vaccines targeting E7 have been shown to cause regression of solid implanted tumors in HPV-16 E6/E7 transgenic mice [131]. These vaccines can also inhibit the growth of thyroid tumors in E6/E7 transgenic mice [132]. Intravaginal immunization with live attenuated *Salmonella enterica* serovar Typhimurium expressing HPV-16 antigens induced transient inflammatory responses in the genital mucosa and conferred protection against subcutaneously implanted HPV-16 tumors [133].

##### Clinical Models

The first clinical *Listeria*-based vaccines, using HPV-16 E7 antigen fused to a fragment of LLO, was found to be well tolerated and did not have any side effects in end-stage cervical cancer patients [134]. However, the trial was a non-controlled study and therefore no inference can be made regarding the differences in overall survival. Further data and trials need to be done to confirm the use of *Listeria*-based vaccines as an efficient vaccine for HPV infection. Although a promising candidate for many cancer vaccines, *Salmonella* has yet to enter clinical trials for therapeutic HPV vaccine.

#### 4.1.2. Viral Vectors

Many recombinant viral vectors (such as adenoviruses, fowlpox viruses, vaccinia viruses, vesicular stomatitis viruses and alphaviruses) have been used for therapeutic HPV vaccine development due to their high infection efficiency and expression of antigens in the infected cells (reviewed in [135]).

##### Preclinical models

A replication-deficient adenovirus encoding fusion proteins comprised of calreticulin, known to enhance MHC-I expression on cell surface, and E7 antigen (CRT/E7) has been shown to generate a potent and protective cellular response to E7, in an established tumour model in mice [136]. This study thus suggested a therapeutic potential for viral vector.

##### Clinical Models

Vaccinia virus is another viral vector that has been tested in both preclinical and clinical trials [135]. Phase I/II clinical trials with recombinant vaccinia virus expressing HPV-16/-18 E6/E7 fusion protein (TA-HPV) have been shown to induce therapeutic effects in patients with early- or late-stage cervical cancer, vaginal intraepithelial neoplasia (VAIN) [137] and VIN (reviewed in [135]. Furthermore depending on the dose and schedule, the antigen-specific immune responses in E6/E7 transgenic mice can be augmented by co-expression of IL-12 in a Semliki Forest virus (SFV), an alphavirus, suggesting that the viral vectors can be further modified to enhance their potency [138]. In a recent phase II clinical trial, subcutaneous injections of TG4001 vaccine, consisting of an attenuated recombinant vaccinia virus containing the sequence for modified HPV-16 E6 and E7, and human IL-2 gene, induced the regression of CIN2/3 lesions in seven of 10 patients [139]. A second phase II clinical trial of randomized vs placebo patients (with a sample size of 200 individuals) is currently ongoing for this vaccine. Vaccinia virus is therefore a promising vaccine candidate against HPV infection. Viral vaccines are moreover safer than DNA vaccines (that will be discussed later in Section 4.3), as they contain only a small fraction of pathogenic genes [135], and they are currently use in both preclinical models and clinical trials.

### 4.2. Protein and Peptide-Based Vaccines

Protein and peptide-based vaccines are the most popular form of HPV therapeutic vaccines. Vaccination with HPV antigenic peptides involves the uptake and presentation of the peptide antigen in association with MHC molecules by DC. The polymorphic nature of MHC molecules in genetically diverse populations makes it difficult to identify one immunogenic epitope which would cover all individuals. However, the use of overlapping, long peptide vaccines based on HPV E6/E7 antigens has been effective in generating antigen-specific T-cell responses. This limited use of peptides is reduced when the entire protein is used. Since protein antigens can be processed by the patient's DC, which contain the relevant human leukocyte antigens (HLA), vaccines based on protein antigens can evade the limitation of MHC specificity associated with peptide vaccines. Adjuvants and fusion with immunostimulatory molecules, such as IL-2, are often used to overcome poor immunogenicity associated with protein and peptide-based vaccines. Several protein and peptide vaccines against HPV E6 and/or E7 have been successfully tested in preclinical and clinical models.

#### Preclinical models

In a E7-expressing TC-1 tumor model, mice were vaccinated subcutaneously with HPV-16 E7 peptide, together with a pan HLA-DR epitope (PADRE) peptide and the TLR3 ligand poly(I:C) adjuvant. PADRE peptide and poly (I:C) were used to enhance the activation of CD4+ T helper cells and dendritic cells, respectively, which altogether generated anti-tumor effects against TC-1. This therapeutic effect was further enhanced when the vaccine was administered in the tumour mass itself, generating higher frequency of E7-specific CD8+ T-cells and leading to a better survival [140].

Like peptides, proteins have been tested as candidates for therapeutic HPV vaccines. To enhance the efficacy of HPV protein-based vaccines, adjuvants such as liposome-polycationic–DNA (LPD) [141] and saponin-based adjuvant [142] have been successfully used in the mouse models. Similarly, fusion of HPV-16 E7 protein with *Bordetella pertusis* adenylyl cyclase (CyaA), which targets proteins to APC [143], or with *Pseudomonas aeruginosa* exotoxin A, which facilitates their translocation to enhance MHC class I presentation [144], have led to improved CTL responses.

#### Clinical models

Several trials have demonstrated the successful use of peptide/protein based vaccines against HPV. A phase I trial involving overlapping HPV-16 E6 and E7 peptides with Montanide ISA51 adjuvant in end-stage cervical cancer patients and VIN grade III patients elicited broad IFN-γ associated T-cell responses [145]. In a similar study, vaccination with long synthetic peptides of E6 and E7 of HPV-16 was effective in the majority (79%) of grade 3 VIN patients [37]. Regarding protein-based vaccines, a HPV fusion protein composed of HPV-6 L2 and E7 (TA-GW) has been shown to be effective in generating antigen-specific T-cell responses in 24 patients with genital warts [146]. A formulation of a vaccine with ISCOMATRIX adjuvant and the HPV-16 E6/E7 fusion protein significantly enhanced E6 and E7-specific CD8+ T cell responses in patients compared with placebo controls [142,147]. Another fusion protein vaccine formulation with of HPV-16 E7 and *Mycobacteria bovis* derived heat shock protein (HSP), known to enhance CTL responses, was used in patients with high-grade anal intraepithelial neoplasia (AIN) [148] and showed clinical responses in 13 out of 38 CIN3 patients [149]. Finally one other successful example is the TA-CIN vaccine (Tissue Antigen - Cervical Intraepithelial Neoplasia), which is a fusion of HPV16 viral proteins L2, E6 and E7. This vaccine is under license from Xenova Research Ltd. (Cambridge, UK) for the treatment of HPV16-associated genital diseases. A recent phase II trial investigating the use of imiquimod/TA-CIN vaccine in patients with VIN demonstrated significant infiltration of CD4 and CD8 T-cells, and complete lesion regression in 63% of patients [123]. The use of peptide/protein-based vaccine against HPV have been shown to be well-tolerated by patients, safe, with no obvious signs of toxicity other than a few flu-like syndromes. They overall have a good immunogenicity and have lead to successful results.

### 4.3. Nucleic Acid-Based Vaccines

#### 4.3.1. DNA Vaccines

DNA-based vaccines are another candidate for HPV-vaccine. They have been extensively studied in preclinical models, in order to optimize the cell targeting, DNA uptake and processing, presentation to MHC molecules...

##### Preclinical models

Strategies to deliver DNA directly into DC may vary. The strategy using Gene gun can efficiently deliver DNA to Langerhans cells in the epidermis, which then express the antigens, become mature and migrate to the draining lymph nodes, where they prime naïve T cells. This method has been shown to elicit HPV antigen-specific T cell immunity and antibody responses [150].

As with protein-based vaccines, DNA encoding HPV antigens are fused with molecules such as FMS-like tyrosine kinase 3 (flt3) ligands [151] and HSP [152] that are capable of targeting the antigens to the DC. Furthermore, DNA construct encoding a fusion protein that links antigenic peptide to MHC class I and β2-microglobulin [153] have been made to enhance antigen presentation by MHC class I molecules. In the similar fashion, DNA construct encoding a fusion protein that links antigenic peptide to a signal peptide for the endoplasmic reticulum and sorting signal for transmembrane proteins [154] have been made to enhance antigen presentation by MHC class II molecules.

In a recent study, a DNA vaccine encoding herpes simplex virus type 1 (HSV-1) glycoprotein D genetically fused to human HPV-16 oncoproteins E5, E6, and E7, induced antigen-specific CD8+ T-cell responses and conferred preventive resistance to transplantable murine TC-1 tumor cells [155]. Co-administration of DNA vectors encoding a co-stimulatory signal, such as GM-CSF or IL-12, has been shown to enhance the therapeutic antitumor effects [155].

In another study, a DNA vaccine encoding calreticulin (CRT) linked to human HPV-16 E7 (CRT/E7) showed increased intercellular uptake and processing of the DNA. With the TLR7 agonist Imiquimod, this vaccine generated E7-specific antitumor effects and prolonged the survival in treated mice, probably by also decreasing the number of myeloid-derived suppressor cells (MDSC) in the tumor microenvironment of tumor-bearing mice [71]. This CRT/E7 vaccine efficiency can be increased by the co-administration of a demethylating agent, 5-aza 2 deoxycytidine (DAC). This treatment decreases the methylation of the DNA and thus inhibit its gene silencing [156].

##### Clinical models

Several DNA vaccines have been tested in clinical trials for CIN2/3 patients (reviewed in [135]). A recent phase-I trial using HPV-16 E7 DNA linked with *M. tuberculosis* HSP70 was tested in CIN2/3 patients. Fifteen CIN2/3 patients were given three different doses of vaccine (three each at 0.5 mg and 1 mg, nine at 3 mg). The vaccine led to complete regression of the lesions in three of nine patients treated with highest dose of the vaccine. E7-specific CD8+ T cell responses were detected in patients treated with vaccine at 1 mg and 3 mg [135,157].

DNA-based vaccines are stable and easy to produce. They can lead to sustained cellular antigen expression compared to protein-based vaccines, which makes them a strong candidate for therapeutic HPV vaccines. However, they have limited potency to invoke innate defence mechanism because of lack of intrinsic ability to amplify and spread *in vivo*. Their use has been shown to be well-tolerated by patients, quite safe, and good immunogenes. Nevertheless, more studies are needed to further improve the efficiency, safety, and cost of potential DNA vaccines.

#### 4.3.2. RNA Vaccines

RNA replicons are vaccines based on RNA viruses. They have the ability to self replicate in the infected host cell. Therefore, they can sustain the cellular antigen expression and as a result, can produce more antigenic protein than conventional DNA vaccines. However unlike DNA vaccines, RNA replicons cannot reproduce by themselves and therefore, can circumvent the possibility of integration into the host genome and cellular transformation. RNA replicons are customized to lack the viral structural genes and thus, the vaccines may be repeatedly administrated in patients without the generation of neutralizing antibodies against viral capsid proteins.

A DNA-launched RNA replicons vaccine, called “suicidal vaccine” has been made to increase the stability of RNA replicons. In this vaccine, suicidal DNA is transcribed into RNA replicons and cells uptaking the suicidal DNA vector eventually die due to apoptosis, thereby minimizing the concerns associated with potential integration of vaccine DNA into the host genome and cell transformation. However, the expression of inserted genes in these vectors is transient and it can shorten the functional lifespan of DC if DNA is targeted to DC, thereby reducing their effectiveness in stimulating the immune system. In one of the preclinical models, a suicidal DNA vector, pSCA1, encoding HPV-16 E7 was fused with BCL-xL, an antiapoptotic protein, to enhance the survival of APC [158]. This vector generated higher E7-specific CD8+ cells and better anti-tumor immune responses than pSCA1 DNA containing E7 gene alone [158]. In another study, vaccination of mice with DNA-launched KUN replicons, a flavivirus which does not induce apoptosis, encoding E7 epitopes generated CTL responses and protected mice against challenge with an E7-expressing epithelial tumor [159]. RNA replicons thus show promising results in preclinical models of HPV infection, but have not yet been investigated in clinical trials.

### 4.4. Whole Cell Vaccines

#### 4.4.1. Tumor Cell-Based Vaccines

Tumor cell-based vaccines have the advantage to cover a broad spectrum of tumor antigens. Tumor cells can be isolated from patients and manipulated to express immunostimulatory proteins such as IL-2, IL-12 and GM-CSF, *ex vivo* to enhance their immunogenicity (for review, see [160]). However, there is a potential concern of introducing new cancers to the patients.

In a recent preclinical study in mice, forced expression in tumors of LIGHT, a ligand for the lymphotoxin-beta receptor, resulted in an increased expression of IFN-γ and chemoattractant cytokines such as IL-1α, MIG, and MIP-2. This correlated with an increased frequency of tumor-infiltrating CD8+ T cells and eradication of large well-established tumors [161].

These vaccines are mainly used when tumor antigens are not identified; however they may not be relevant in case of HPV as tumor antigens associated with HPV-infection are largely known and this could be the reason that these vaccines have not yet tested in clinical trials for HPV-associated cancers.

#### 4.4.2. Dendritic Cell-Based Vaccine

DC vaccines are another way to enhance T-cell mediated immunity against HPV-associated lesions.

##### Preclinical models

DC-based vaccines are the preferred choice for vaccine development and a number of methods have been used including the usage of different vectors, pulsing of DC with proteins and peptides or transfecting DC with DNA or RNA (see above and reviewed in [135]). In a TC-1 murine tumor model, vaccination with E7-presenting DC transfected with anti-apoptotic siRNA targeting Bim was shown to generate E7-specific CD8+ T cells and decrease tumor growth [162].

##### Clinical Models

In humans, autologous DCs were pulsed with HPV16 or HPV18 E7 recombinant proteins and E7-specific CD8+ T cell responses were observed in four out of 11 late stage cervical cancer patients [163]. In another clinical study, stage IB or IIA cervical cancer patients were vaccinated with autologous DC pulsed with recombinant HPV16/18 E7 antigens and keyhole limpet hemocyanin (KLH), an immunological carrier protein. This vaccine generated E7-specific T cell responses in 8 out of 10 patients and antibody responses in all patients [164].

DC vaccines are patient-specific, where clinicians harvest DC from the patient, load them with tumor antigens such as E6 or E7, and then inject these DC back into the patient, where they elicit antigen-specific potent antitumor immune response. The success of Provenge^®^, a DC vaccine incorporating prostatic acid phosphatase, in patients with advanced prostate cancer has generated strong interest for DC-based vaccines [165,166]. However, DC-based vaccines have serious limitations. Since these vaccines cannot be produced at a large scale, they are labour intensive and expensive. Nevertheless, DC-based vaccines have been tested in patients with HPV-associated cervical cancer by successfully transducing genes coding for E6 and E7 into DC.

### 4.5. Combinational Approaches

In Section 4 we discussed the different formulations that can potentially been used to create a therapeutic vaccine. In the examples we cited, some of the studies already used combinational approaches, by adding an adjuvant or a co-stimulatory signal to the vaccine itself. In this paragraph, we just want to highlight it a bit more.

Prime-boost vaccination strategies use several of the available therapeutic vaccines in combination to induce higher levels of tumor specific immune response. Some of these have been evaluated in clinical trials for therapeutic HPV vaccines. In one trial, nine patients developed HPV16 specific T cell responses and three out of 10 patients showed a significant reduction in the size of the lesion, when high grade VIN were primed with TA-HPV and boosted with TA-CIN [137].

Therapeutic HPV vaccines may potentially be combined with other therapeutic methods such as radiotherapy and chemotherapy to enhance the clinical outcome. In an experimental model, treatment with low-dose radiotherapy made the TC-1 tumor cells more susceptible to lysis by E7-specific CD8+ T cells, and enhanced antitumor effects in tumor bearing mice [167]. In a similar fashion, combination of 5,6-dimethylxanthenone-4-acetic acid (DMXAA), a vascular disrupting agent, and treatment with E7 DNA vaccination generated potent antitumor immune responses in the splenocytes of tumor bearing mice [168].

Therapeutic HPV vaccines may also be combined to co-stimulatory signal delivery or cytokine adjuvantizing. The use of CTLA-4 antibodies has been shown to be effective in murine TC-1 tumor models [169]. More recently, peritumoral administration of IL-12-producing tumor vaccines enhanced the effect of the cytostatic chemotherapeutic agent, gemcitabine, which was correlated with high production of IFN-γ by splenocytes [170].

Another strategy to improve HPV-vaccine is to combine them with the depletion of regulatory cells such as Treg, MDSC, or NKT cells, as discussed in Section 2. However in a recent study, depletion of Treg did not enhance the immune response induced by SFV-based HPVE6/E7 vaccine against murine tumors, suggesting that the SFVeE6/7 vaccine may not require additional immune interventions [171].

Thus, as it has already been described in many other cancer types, combinational approaches might be the gold strategies to generate a successful anti-HPV therapeutic vaccine.

## 5. HPV Immunotherapy, the Future

Although preventive HPV vaccines are now available to use, the high prevalence of HPV-associated malignancies worldwide suggests a potential benefit from developing therapeutic HPV vaccines. New leads, news targets and new research axis have to be investigated to maximize the chances to find a way towards therapeutic vaccines.

### 5.1. Future Challenges and Resources?

The HPV-derived VLP obtained by the self assembly of the viral L1 capsid protein have been generated in yeast and insect cell lines. However, the development of contained plant systems (*i.e.*, plant cell suspension, hairy root cultures, microalgae, etc) provides a powerful alternative for the production of recombinant therapeutic molecules, such as IgG, IL-12 or IFNs. Plant cell suspension can be derived from tobacco, rice, soybean, tomato, alfalfa and carrot plants; hairy root cultures are generated from the interaction between *Agrobacterium rhizogenes*, a Gram-negative soil bacterium, and a host plant; and microalgae are photosynthetic heterotrophic microorganisms found in wet environments [172].

The advantages offered by these systems are firstly their low-cost production, the safety of their production, their ability to generate post-translational modifications and to synthesize folded or assembled protein multimeres correctly, as well as alleviating ethical issues in generating high-grade pharmaceuticals. On the other hand, some parameters still need to be improved, as the protein yield is quite low (0.01–0.2 g/L) compared to mammalian or eukaryotic systems (1–3 g/L or 0.5–5 g/L, respectively), and also some post-translational modifications need to be adapted [173].

There is recent evidence of the successful usage of plant-produced pharmaceuticals by Biolex Therapeutics (www.biolex.com). The results of this phase II clinical trial for the treatment of patients with chronic hepatitis C with Locteron, an alpha-IFN produced in duckweed, were published in March 2011 [174]. In relation to vaccine development, the very first veterinary vaccine derived from tobacco cell culture has been engineered by Dow AgroSciences (www.dowagro.com/), obtaining US Department of Agriculture (USDA) approval in 2006. Although not yet available commercially, this novel product represents the first approval for a plant-derived vaccine.

### 5.2. Conclusions

**Figure 2.**
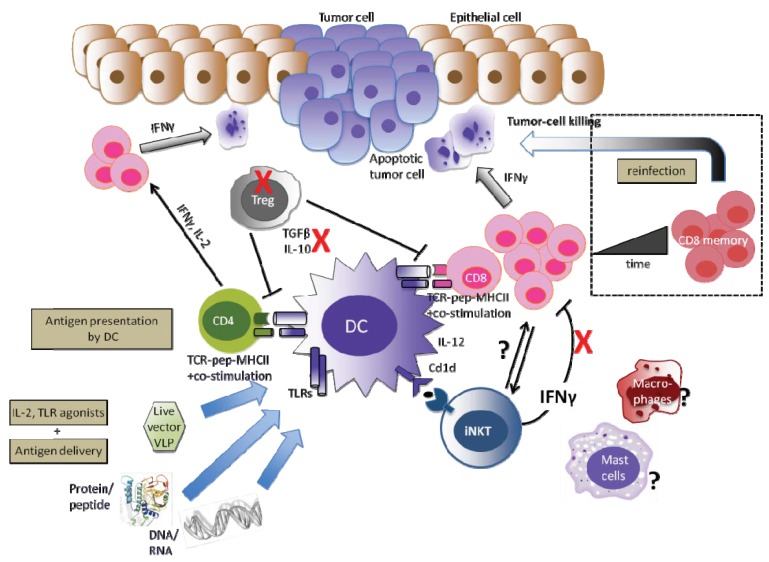
Therapeutic strategies against HPV-infected lesions. The tumor microenvironment is composed of cells of the adaptive immune system (such as CD4 and CD8 T-cells, Treg) and cells of the innate immune system (such as DC, NKT, macrophages) and potentially other cells (Mast cells? MSDCs?) that could have a role in the response to HPV. Soluble factors including regulatory cytokines IL-10, TGF-β or IFN-γ may also be involved. This figure provides an overview of the different strategies that can be employed to generate therapeutic effects against HPV-infected epithelial lesions, which include live vector (viral/bacterial), protein or peptide, or nucleic acid (DNA/RNA) or VLP, together with the use of adjuvants such as TLR agonists or cytokines. Overall, the ideal vaccine would activate effector killer T-cells while silencing regulatory factors. The generation of memory cells that can mount a faster and stronger immune response would prevent reinfection by HPV and cancer relapse (inset).

A successful HPV therapeutic vaccine should deal with both arms of the innate and adaptive immunity. The ideal vaccine should therefore generate high numbers of efficient tumor-specific and cytotoxic effector T-cells and promote inflammation. Together, this would prevent persistent HPV infection from progressing towards cervical cancer or even eradicate HPV. Moreover, within the tumor microenvironment and as already proven in other cancers, there are many cells and factors that may inhibit T-cell effector function and hinder the success of effective immunotherapy, such as Treg cells and immunosuppressive cytokines such as IL-10 and TGF-β. Similarly, IFN-γ released by iNKT cells has shown to be immunosuppressive in the E7-expressing skin. Therefore, the transient depletion of Treg, the blocking of IL-10 and TGF-β, the fine control of NKT-derived IFN-γ in the tumor microenvironment may enhance therapeutic HPV vaccine potency. More studies and knowledge are required to determine the role of those cells, as well as MSDC, mast cells and macrophages and other regulatory components of the innate immune system, that compose the tumor microenvironment and could play a role in HPV-associated cancers (summarized in Figure 2).

A potent therapeutic vaccine will most likely require the combination of current delivery systems (VLP, live vector, protein and DNA/RNA, plant-derived pharmaceuticals) associated with conventional therapeutic approaches (chemotherapy/radiotherapy, targeted depletion). With the increasing discovery of new drugs, development of new adjuvants, and the better understanding of tumor biology, we will have more opportunities to develop improved therapeutics against HPV-associated cancers. Successful clinical trials for therapeutic HPV vaccines based on positive preclinical data have now been published [37,123], and show that curing HPV-associated lesions is feasible.

We thank Graham Leggatt, Fiona McMillan and James Wells for critical review of the manuscript. This work was supported by funding from the National Health and Medical Research Council (Program Grant No. 569938) and the NIH grant No. 5U01CA141583, Cancer Council Australia, Cancer Research Institute (New York), Lions Medical Research Foundation, Australian Cancer Research Foundation and a Queensland Government Premiers Fellowship. IHF derived royalty income from prophylactic vaccines against cervical cancer based on HPV VLP technology.

## Abbreviations

APC: Antigen presenting cells
CTL: Cytotoxic T lymphocytes
DC: Dendritic cells
HIV: Human Immunodeficiency virus
HPV: Human Papillomavirus
NKT: Natural killer T cells
Treg: Regulatory T cells
VLP: Virus-like particles
